# Chromosomal-scale genome assembly of the Mediterranean mussel *Mytilus galloprovincialis*

**DOI:** 10.1038/s41597-024-03497-5

**Published:** 2024-06-17

**Authors:** Guo-dong Han, Dan-dan Ma, Li-na Du, Zhen-jun Zhao

**Affiliations:** College of Life Science, Yantai University, Yantai, Shandong 264005 China

**Keywords:** Genomics, Genome

## Abstract

The Mediterranean mussel, *Mytilus galloprovincialis*, is a significant marine bivalve species that has ecological and economic importance. This species is robustly resilient and highly invasive. Despite the scientific and commercial interest in studying its biology and aquaculture, there remains a need for a high-quality, chromosome-scale reference genome. In this study, we have assembled a high-quality chromosome-scale reference genome for *M. galloprovincialis*. The total length of our reference genome is 1.41 Gb, with a scaffold N50 sequence length of 96.9 Mb. BUSCO analysis revealed a 97.5% completeness based on complete BUSCOs. Compared to the four other available *M. galloprovincialis* assemblies, the assembly described here is dramatically improved in both contiguity and completeness. This new reference genome will greatly contribute to a deeper understanding of the resilience and invasiveness of *M. galloprovincialis*.

## Background & Summary

The Mediterranean mussel, *Mytilus galloprovincialis* Lamarck 1819, is a gregarious species that attaches to rocks or other hard surfaces using byssal threads. The mussel plays a crucial ecological role as an ecosystem engineer by creating habitat and promoting environmental heterogeneity, thereby enhancing local biodiversity^1^. The mussel is also known to accumulate contaminants in its tissues from the surrounding environment^2–5^. As a result, it has been widely used as a reliable bioindicator in various monitoring programs, such as the Mussel Watch Programme^6^. In addition to its ecological role as an ecosystem engineer and bioindicator, *M. galloprovincialis* also holds considerable commercial value. *M. galloprovincialis* is a widely cultivated bivalve species globally. In 2015, the worldwide production of *M. galloprovincialis* for human consumption exceeded 1.1 million tonnes^7^.

**Fig. 1:**
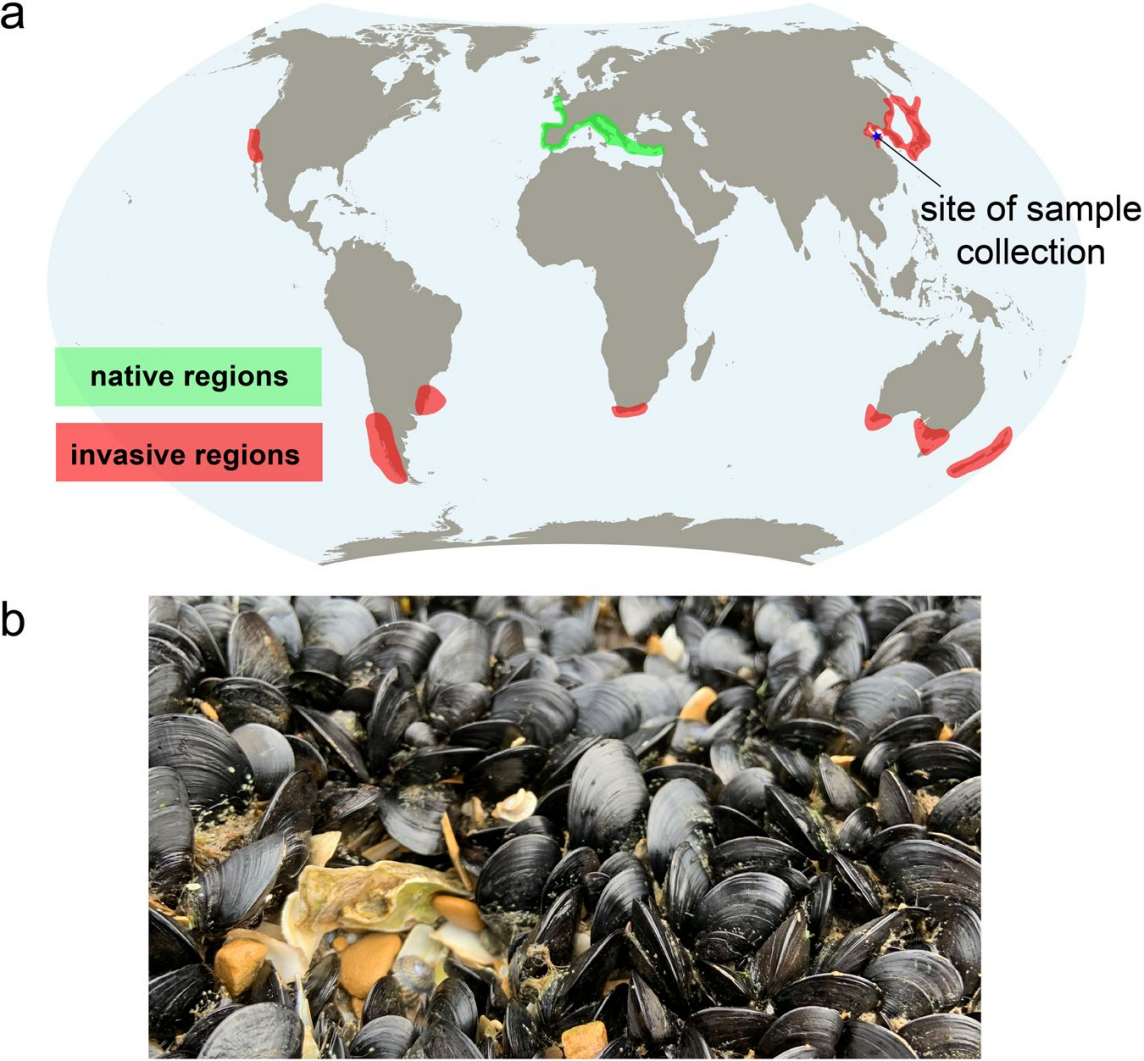
The global distribution of the Mediterranean mussel, *Mytilus galloprovincialis*, is shown in the map (**a**). The green regions indicate the native range, while the red regions indicate the invasive range. The distribution data was compiled from various sources^51–58^. In the photo (**b**), gregarious *M. galloprovincialis* can be seen on a rocky shore in Dalian, China. Photo credit: Guo-dong Han.

The mussel *M. galloprovincialis* is a highly invasive species that originated in the Mediterranean Sea and the eastern Atlantic, extending north to the British Isles. The species has been introduced to different areas via ballast water over the past century, and is now found in temperate coastal regions of both the northern and southern hemispheres (see Fig. 1a for detail). The Global Invasive Species Programme has identified *M. galloprovincialis* as one of the top 100 worst invasive species globally due to its significant impact on biological diversity^8^. The invasive success of *M. galloprovincialis* in central and southern California is believed to be partly attributed to its physiological adaptations that allow it to outperform *M. trossulus* in high temperatures^9^. Fields and colleagues^9^ discovered that a slight alteration in the structure of cytosolic malate dehydrogenases in *M. galloprovincialis* allows them to function effectively at higher temperatures. Recent advancements in genomic screening have enabled the identification of a greater number of genetic variations associated with differences in environmental conditions, such as temperature.

Chromosome-scale reference genomes are essential for applying genomics to biology, aquaculture, and biodiversity conservation. They offer improved contiguity and completeness compared to fragmented genome assemblies, enabling the testing of crucial ecological and evolutionary hypotheses. Although there is significant scientific and commercial interest in mussels for both biology and aquaculture purposes, the availability of a high-quality, chromosome-scale reference genome for *M. galloprovincialis* is currently limited^10–12^. The genome of *M. galloprovincialis*, similar to other bivalves, is relatively large and complex, and frequently exhibits high heterozygosity^13^. Particularly, the genome of *M. galloprovincialis* exhibits high levels of hemizygosity (only one of the two chromosomal pairs encodes a region or block of DNA) compared to other molluscs^11,14^. Previous attempts at assembly of this species were significantly hindered by these factors.

**Fig. 2:**
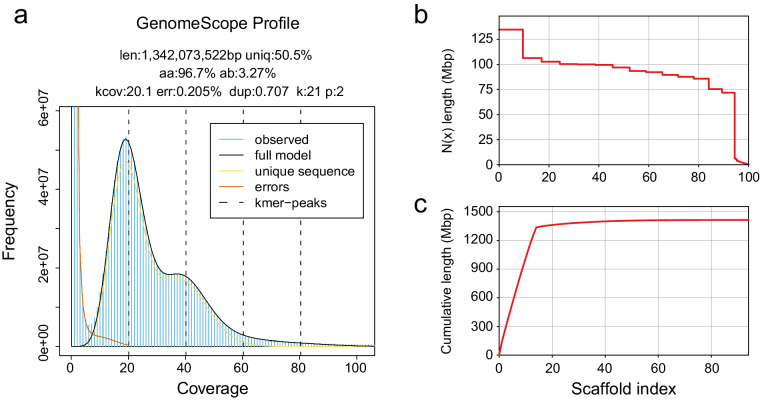
Characteristics of the *M. galloprovincialis* genome. (**a**) The k-mer spectra output is generated by *GenomeScope* 2.0 using short reads obtained from whole-genome sequencing (WGS). (**b**) N(x) plots illustrate the contiguity profile of the primary assembly. (**c**) The cumulative length of the primary assembly is plotted for all scaffolds.

**Fig. 3:**
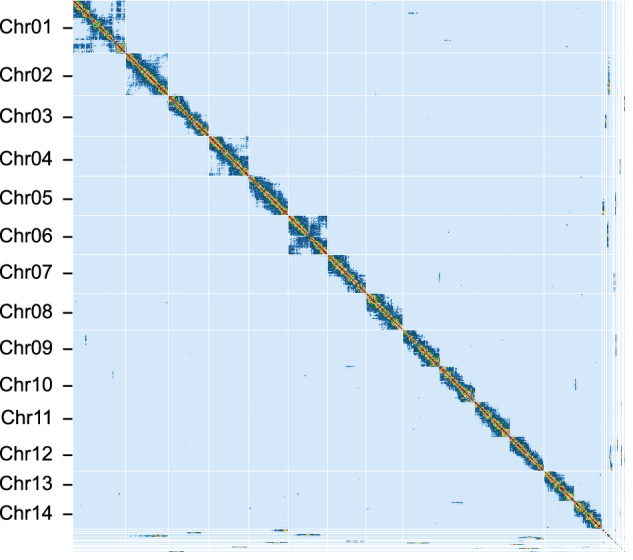
Hi-C contact maps for the primary genome assembly. Hi-C contact map represents the spatial proximity of genomic regions in a linear format. Each cell in the contact map contains sequencing data that confirms the connection between two specific regions. The scaffolds are arranged in order of length and are visually separated by white lines.

In this study, we used PacBio HiFi technology to sequence and assemble the genome of *M. galloprovincialis*. We also utilized high-throughput chromosome conformation capture (Hi-C) technology to achieve chromosome-scale scaffolding. As a result, we constructed a high-quality chromosome-scale genome assembly for *M. galloprovincialis*. The primary assembly is highly continuous, complete, and accurate. Key metrics include a scaffold N50 of 96.9 Mb (Fig. 2b), k-mer completeness of 68.8, and a k-mer-based quality value (QV) of 51.1 (Table 1). Gene annotation analysis using the metazoa_odb10 lineage dataset showed a completeness of 97.5%, indicating high annotation quality. Certain taxonomically restricted genes were not identified during the BUSCO assessment of genome completeness. For instance, *myticin* was not part of the gene set in metazoa_odb10, but it was found in the genome. Compared to the other available *M. galloprovincialis* assemblies in GenBank, our assembly significantly improves contiguity and functional completeness (as measured by the number of complete BUSCO notations). Previous karyometric analysis has shown that *M. galloprovincialis* has 14 chromosomes^15,16^. The application of Hi-C in this study resulted in 14 long scaffolds in the primary assembly, approaching chromosome-level assembly. The Hi-C contact map suggests that the primary assembly is highly contiguous (Fig. 3).

**Table 1:** Statistics of sequencing and assembly.

Genome sequence	PacBio HiFi reads	2 PACBIO_SMRT (Sequel IIe), 2.8 M spots, 53.1 G bases	
	Hi-C Illumina reads	Illumina NovaSeq, 566.3 M spots, 169.9 G bases	
	WGS Illumina reads	Illumina NovaSeq, 216 M spots, 64.8 G bases	
Genome Assembly Quality Metrics	HiFi read coverage	37.9×	
		Primary	Alternate
	Number of contigs	181	20,451
	Contig N50 (bp)	24,237,878	231,291
	Longest contigs (bp)	57,167,429	1,552,391
	Number of scaffolds	94	17,876
	Scaffold N50 (bp)	96,880,329	545,759
	Largest scaffold (bp)	134,701,878	37,314,948
	Size of final assembly (bp)	1,414,122,127	1,566,570,365
	k-mer based QV	51.1	46.8
		Full assembly = 48.4	
	k-mer completeness	68.8	62.3
		Full assembly = 95.4

**Fig. 4:**
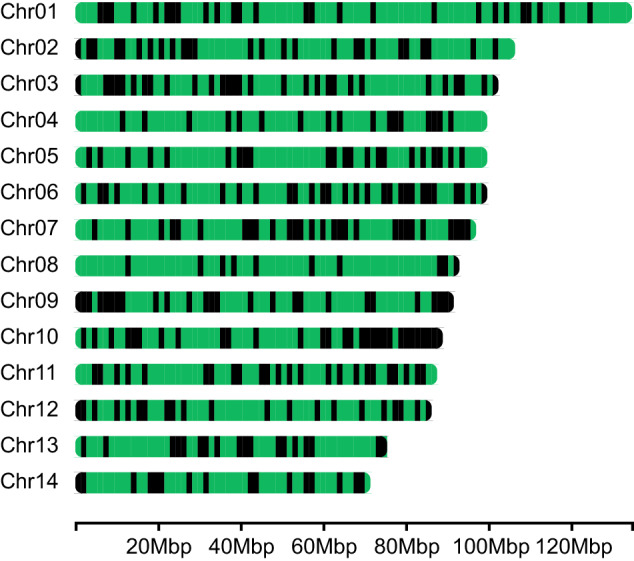
Chromosomal map of hemizygous loci. Deletions at least 10 kb in length that were not associated with tandem repeats were shown in black.

The assembly exhibited widespread hemizygous regions, with 8.79% deletions of the total genome (Fig. 4). A total of 18,429 genes were identified that are located within hemizygous regions. The hemizygosity we observed in this study is higher than that of other molluscs (ranging from 0.17% to 6.69%^14^). However, it is significantly lower than the hemizygosity estimated in a previous study for the same species (36.78% with a genome size of 1.28Gb^11^). The observed differences may be due to the fact that the sample used in our study was collected from the invasive region, whereas the sample used in the previous study was collected from its native region^11^. Furthermore, the genome size of *M. galloprovincialis* could be notably impacted by hemizygous regions. Increased hemizygosity may lead to a reduction in genome size of this species. Therefore, it seems that structural variation, especially large insertion/deletion polymorphisms, play a crucial role in promoting successful invasiveness. Future studies should focus on confirming the significance of structural variation in invasive adaptation by resequencing individuals from a wider range of native and invasive locations.

Our chromosome-level reference genome for *M. galloprovincialis* provides a more complete understanding of its resilience and invasiveness compared to incomplete and fragmented reference genomes. Previous studies using a fragmented reference genome (GCA_001676915.1) identified a significant number of single nucleotide polymorphisms (SNPs) in *M. galloprovincialis*, indicating the presence of standing genetic variation that enables rapid adaptation to ocean acidification^17^. Another study found a large number of SNPs and demonstrated that divergent climatic factors have driven adaptive genetic variation in *M. galloprovincialis* over the past century, contributing to its successful invasion of various thermal habitats^18^. Our complete reference genome will further enhance the identification and annotation of standing genetic variation and adaptive genetic variation in this species. Additionally, the whole genome data will be valuable for the aquaculture industry in developing genetic markers for economically important traits. Genotypes in quantitative trait loci (QTLs) are associated with specific phenotypes, such as shell size and body weight, and can be used as markers for selection in breeding programs^13^. In conclusion, our chromosome-level reference genome, combined with future population genomics studies, will greatly contribute to a deeper understanding of the resilience and invasiveness of *M. galloprovincialis* and facilitate its application in aquaculture.

## Methods

### Biological materials

One female mussel was collected from Yangmadao, Shandong Province, China (37.46342°N, 121.59541°E) on June 1, 2022. The mussel had a shell length of 76 mm. The specimen was subsequently taken to Yantai University, where the adductor muscle was dissected and promptly frozen using liquid nitrogen. The adductor muscle tissue was used to prepare Illumina paired-end library, HiFi SMRTbell library and Hi-C library.

### Nucleic acid extraction, library preparation and sequencing

The genomic DNA was extracted using the QIAGEN Genomic-tip 100/G kit (QIAGEN, Germany) following the manufacturer’s instructions. DNA quality was assessed using pulsed field gel electrophoresis, and DNA concentration and purity were measured using the Qubit DNA Assay Kit (Invitrogen, USA) and Nanodrop 2000 (Thermo, USA), respectively.

A library with an insert size of 500 bp was prepared using the TruSeq Nano DNA Library kit (Illumina, CA, USA) by randomly fragmenting and ligating adaptors to the DNA sequences. Paired-end sequencing with 150 bp was performed using the NovaSeq 6000 sequencing system (Illumina, CA, USA).

For the HiFi SMRTbell library, 8 μg of DNA was used to prepare library using the SMRTbell Express Template Preparation Kit 2.0 (Pacific Biosciences, USA) following the manufacturer’s recommendations. The library was sequenced using the HiFi SMRTbell sequencing method with an average fragment size of 15 to 20 kb. The DNA library was loaded onto the flow cell with sequencing buffer and loading beads, and sequencing was performed using the DNA Sequencing Reagent Kit (Pacific Biosciences, USA) according to the manual.

To construct Hi-C libraries, the adductor muscle was grinded with liquid nitrogen and cross-linked with 4% formaldehyde solution and then quenched with glycine. The cross-linked sample was lysed and the nuclei were isolated. The nuclei were then solubilized and digested with the restriction enzyme MboI. The DNA ends were marked with biotin-14-dCTP and the cross-linked fragments were ligated. The nuclear complexes were reverse cross-linked and the DNA was purified. Non-ligated fragment ends were treated with T4 DNA polymerase to remove biotin. The sheared fragments were repaired and the Hi-C samples were enriched using streptavidin C1 magnetic beads. The Hi-C libraries were prepared by adding A-tails to the fragment ends and ligating Illumina paired-end (PE) sequencing adapters. The libraries were amplified by PCR and sequenced on an NovaSeq 6000 sequencing system.

### Genome assembly

We used *Meryl* v1.3^19^ to generate k-mer counts (k = 21) from Illumina WGS reads. The resulting k-mer database was then utilized in *GenomeScope2.0*^20^ to estimate various genome features, such as sequencing error, genome size, heterozygosity, and repeat content. We estimated the genome size to be 1.34 Gb and the nucleotide heterozygosity rate to be 3.27% (Fig. 2a).

For the assembly of the *M. galloprovincialis* genome, we employed the *de novo* assembler *HiFiasm* v0.19.5-r592^21^. The final output is a diploid assembly comprising two pseudohaplotypes: a primary assembly and an alternate assembly. We used *purge_dups* v1.2.5^22^ to identify duplicated sequences and overlapping contigs in the assemblies. The assemblies were then scaffolded using Hi-C data with *SALSA*^23^. Gaps remaining after scaffolding were closed using PacBio HiFi reads and *YAGCloser* (https://github.com/merlyescalona/yagcloser). Contamination was checked using *BlobToolKit* v2.6.5^24^.

The primary assembly was manually curated using Hi-C contact maps. The Hi-C data was aligned to the reference using *bwa mem* v0.7.17-r1188^25^. Ligation junctions were identified and Hi-C pairs were generated using *pairtools* v1.0.2^26^. A 50 kb Hi-C matrix was created using *cooler* v0.9.2^27^ and balanced using *hicExplorer* v3.6^28^. And *PretextMap* v0.1.9 (https://github.com/sanger-tol/PretextMap) were used for visualizing the contact maps (Fig. 3).

### Structural variation detection

We employed the allelic structural variation detection pipeline^29^ to determine the hemizygous regions of the primary assembly. Tandem repeats in genome assembly were identify using *trf* v4.09^30^. PacBio HiFi reads were aligned to the primary assembly using *minimap2* v2.17-r941^31^. Structural variations in the primary assembly were identified using *pbsv* v2.9.0 (https://github.com/PacificBiosciences/pbsv). Deletions at least 10 kb in length that were not associated with tandem repeats were visualized using *chromoMap* v4.1.1^32^. The primary assembly exhibited widespread hemizygous regions, with 8.79% deletions of the total genome length (Fig. 4). When insertions were taken into account, the rate of hemizygosity of the primary assembly increased to 16.32%.

### Genome annotation

Prior to gene structure annotation, repetitive sequences in the primary assembly were identified and masked. A *de novo* canonical database of repetitive elements was constructed using *RepeatModeler* v2.0.3^33^ with the “-LTRStruct” option. Repeat families specific to Bivalvia were extracted from Repbase (RepeatMaskerEdition-20181026) and Dfam (v3.8) database to create a homology canonical database. These two databases were combined and used with *RepeatMasker* v4.1.2^34^ to identify and classify repeats in the primary assembly. Approximately 60.42% (854 Mb) of the assembled sequences of *M. galloprovincialis* were identified as repetitive sequences (Table 2).

**Table 2:** Statistics of repetitive sequence of *M. galloprovincialis* genome.

	Number of elements	Length occupied (bp)	Percentage of sequence (%)
Retroelements	477,323	137,911,894	9.75
SINEs	167,368	23,154,118	1.64
LINEs	243,107	82,479,875	5.83
LTR elements	66,848	32,277,901	2.28
DNA transposons	496,298	104,219,346	7.37
Rolling-circles	225,643	42,466,079	3.00
Unclassified	1,941,616	453,996,101	32.10
Small RNA	18,551	3,764,216	0.27
Satellites	74,746	13,641,986	0.96
Simple repeats	169,899	8,616,545	0.61
Low complexity	29,221	1,383,287	0.10
Masked		854,358,484	60.42

Protein-coding genes were identified using a combination of ab initio prediction and transcriptome-assisted methods. The *BRAKER2* v2.1.6^35^ gene prediction pipeline was used to predict genes from repeat-masked genome sequences. Transcriptome-assisted annotation was performed using downloaded Illumina RNA-Seq data from seven tissues (PRJNA230138). The RNA-Seq data were aligned to the primary assembly using *HISAT2* v2.2.1^36^. The resulting BAM files were merged and used for genome-guided assembly of transcripts using *Trinity* v2.1.1^37^. The genome-guided RNA-Seq assemblies were then inputted to *PASA* v2.5.2^38^ for transcriptome database generation. Finally, ab initio gene predictions and transcript alignments were combined into weighted consensus gene structures using *EVidenceModeler* v1.1.1^39^. Finally, we identifying 58,480 genes in the primary assembly (Table 3). We identified protein-coding regions located within hemizygous regions using *bedtools* intersect v2.30.0^40^. Genes located within hemizygous regions were determined by identifying at least one exon falling within these regions. A total of 38,301 exons were found to be located within hemizygous regions, and these exons are associated with 18,429 genes.

**Table 3:** Statistics of functionally annotated genes of *M. galloprovincialis* genome.

		Number of genes	Percentage (%)
Protein-coding genes		58,480	
Databases	Nr	54,374	93.0
	UniProt	28,043	48.0
	GO	23,011	39.3
	KEGG	9,547	16.3

To perform functional annotation, we compared the predicted protein sequences to the Nr and UniProt database using *blastp* v2.13.0^41^ with an e-value threshold of 1e-5. Additionally, we used *InterProScan* v5.57^42^ and *kofam_scan* v1.3.0 (https://github.com/takaram/kofam_scan) for Gene Ontology (GO) and Kyoto Encyclopedia of Genes and Genomes (KEGG) annotation, respectively. Among the predicted proteins, 54,374, 28,043, 23,011, and 9,547 proteins were matched to the Nr, Uniprot, Go, and KEGG databases, respectively (Table 3).

## Data Records

The raw sequence data reported in this paper have been deposited in the Genome Sequence Archive^43^ in National Genomics Data Center (NGDC)^44^, China National Center for Bioinformation/Beijing Institute of Genomics, Chinese Academy of Sciences (GSA: CRA015597^45^) that are publicly accessible at https://ngdc.cncb.ac.cn/gsa. The sequence accessions for the primary and alternate assemblies have been deposited in the NCBI, under accession number JAWDJN000000000^46^ and JAZKRD000000000^47^, respectively. The genome annotation file, functional annotations for predicted genes, bed files of hemizygous loci and genes located within hemizygous regions are available in the figshare repository, respectively^48,49^.

## Technical Validation

**Fig. 5:**
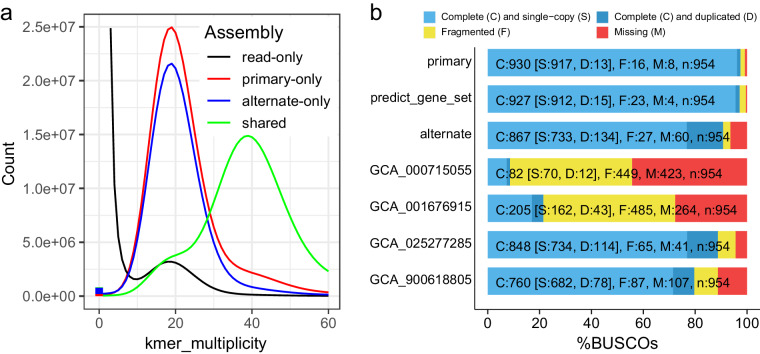
Quality assessment of assembled genome of *M. galloprovincialis*. (**a**) Merqury assembly spectrum plots for evaluating k-mer completeness. (**b**) Comparison of BUSCO scores (metazoa_odb10) for the assemblies of *M. galloprovincialis* with four other assemblies accessible in the NCBI database.

We used the QV pipeline of *Merqury*^19^ to estimate the assembly QV based on k-mer analysis. The script “*best_k.sh*” in *Merqury* was employed to determine the optimal k-mer length. *Meryl* was then utilized to calculate the number of k-mers in the Illumina WGS reads using default settings. The QV evaluation was performed in *Merqury* using the output from *Meryl* and the assembly. The findings demonstrated a k-mer completeness of 68.8 and a k-mer-based QV of 51.1, suggesting high levels of completeness and accuracy. The spectra-asm plot illustrates a well-assembled diploid genome (Fig. 5a), with 1-copy k-mers unique to the primary assembly (red) and alternate assembly (blue), and 2-copy k-mers shared by both assemblies (green).

To evaluate the accuracy of the assemblies and the predicted gene set, we employed *BUSCO* v5.2.0^50^ with metazoa_odb10 to determine standard metrics of assembly completeness. Gene annotation analysis revealed a completeness rate of 97.5%, signifying high-quality annotation. The ultimate predicted gene set comprised 58,480 genes with a BUSCO value of 97.2%. Our assembly demonstrated superior contiguity and functional completeness compared to other *M. galloprovincialis* assemblies available in GenBank (Fig. 5b).

## Data Availability

In this study, software tools were utilized as described in the Method section. All bash command lines and scripts are available at the GitHub repository: https://github.com/HanLab2018/Mytilus-galloprovincialis-genome-assembly.
